# Drug Therapies for the Management of Sickle Cell Disease

**DOI:** 10.12688/f1000research.22433.1

**Published:** 2020-06-11

**Authors:** Parul Rai, Kenneth I. Ataga

**Affiliations:** 1 Department of Hematology, St. Jude Children's Research Hospital, Memphis, TN, USA; 2 Center for Sickle Cell Disease, University of Tennessee Health Science Center, Memphis, TN, USA

**Keywords:** Sickle cell disease, Treatment, Novel Drugs, Drug Development, Clinical Trials

## Abstract

Sickle cell disease (SCD) afflicts millions of people worldwide but is referred to as an orphan disease in the United States. Over the past several decades, there has been an increasing understanding of the pathophysiology of SCD and its complications. While most individuals with SCD in resource-rich countries survive into adulthood, the life expectancy of patients with SCD remains substantially shorter than for the general African-American population. SCD can be cured using hematopoietic stem cell transplantation and possibly gene therapy, but these treatment approaches are not available to most patients, the majority of whom reside in low- and middle-income countries. Until relatively recently, only one drug, hydroxyurea, was approved by the US Food and Drug Administration to ameliorate disease severity. Multiple other drugs (L-glutamine, crizanlizumab, and voxelotor) have recently been approved for the treatment of SCD, with several others at various stages of clinical testing. The availability of multiple agents to treat SCD raises questions related to the choice of appropriate drug therapy, combination of multiple agents, and affordability of recently approved products. The enthusiasm for new drug development provides opportunities to involve patients in low- and middle-income nations in the testing of potentially disease-modifying therapies and has the potential to contribute to capacity building in these environments. Demonstration that these agents, alone or in combination, can prevent or decrease end-organ damage would provide additional evidence for the role of drug therapies in improving outcomes in SCD.

## Introduction

Although referred to as an orphan disease in the United States (US), sickle cell disease (SCD) affects millions of individuals worldwide, with the vast majority residing in sub-Saharan Africa and India ^1^. SCD is characterized by the presence of sickle hemoglobin (HbS), hemolytic anemia, vaso-occlusive complications, and cumulative end-organ damage. The mortality rate associated with SCD in sub-Saharan Africa remains high, with an estimated 50 to 90% of children dying before the age of 5 ^2^. However, the majority of children with SCD in resource-rich countries live to adulthood ^3– 5^. Despite increased survival to adulthood, individuals with SCD in resource-rich nations continue to have a shorter life expectancy than the general population ^6– 9^. There has been substantial progress with the use of allogeneic bone marrow transplantation as a curative therapy in SCD, and increasing evidence supports the curative potential of gene therapy and gene editing ^10, 11^. However, as these modalities are not available to the vast majority of patients, most of whom reside in resource-limited countries, the availability of drug therapies that are safe, effective, and affordable remains highly desirable.

This review will focus on approaches to develop drug therapies in SCD, ongoing and recently completed trials, and our perspective on the use of approved drugs.

## Pathophysiology

**Figure 1.**
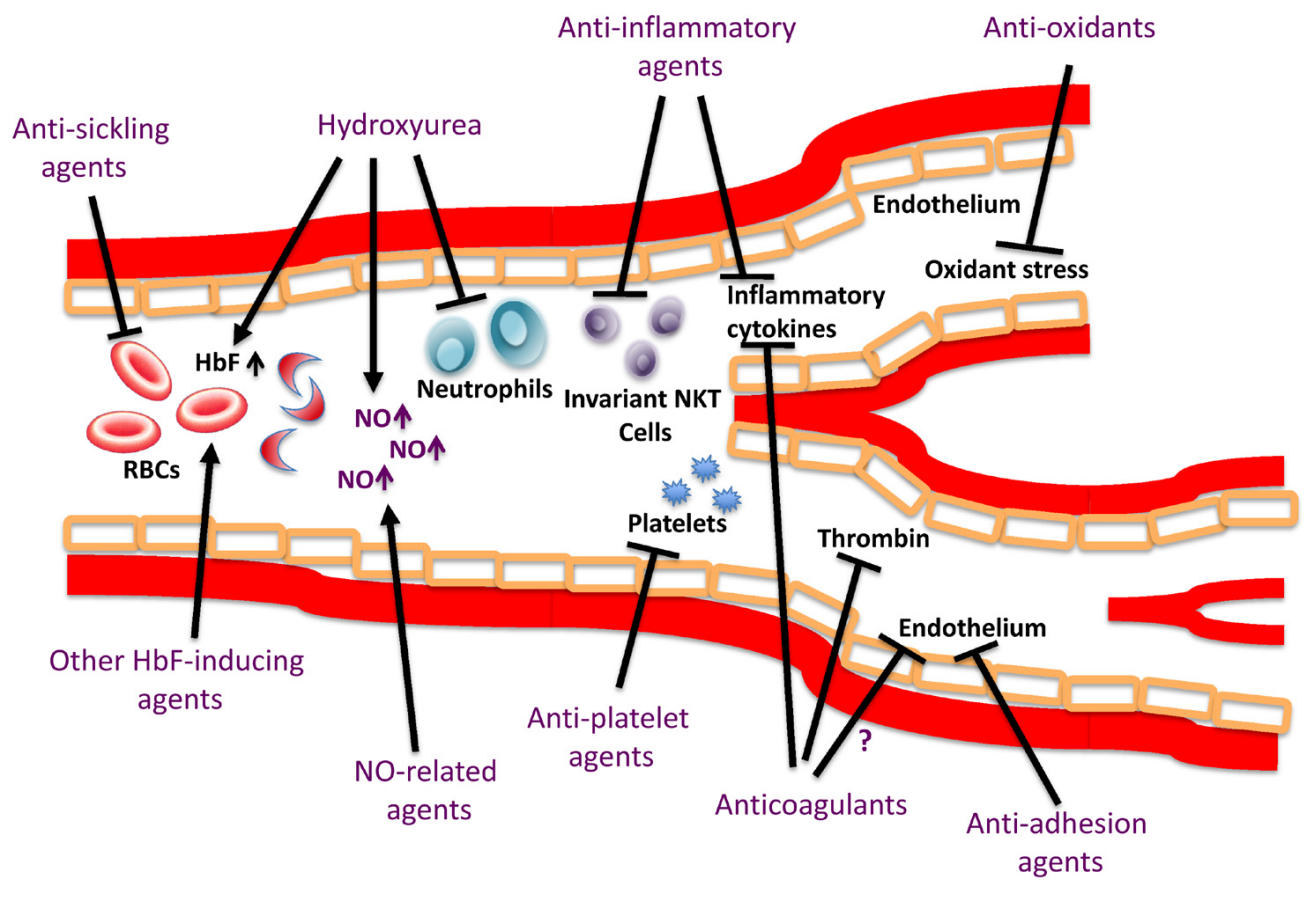
Potential targets of pharmacologic agents in sickle cell disease HbF, hemoglobin F; NKT, natural killer T-cells; NO, nitric oxide; RBCs, red blood cells. Adapted with permission from Ataga KI and Stocker J. *Br J Haematol*, 2015.

The development of effective therapies for SCD depends on an adequate understanding of its pathophysiology. Although the pathophysiology of SCD is complex and involves multiple pathways, the primary event is due to the polymerization of HbS following deoxygenation ^12^. The rate and extent of polymer formation depends on the degree and duration of HbS deoxygenation, presence of fetal hemoglobin (HbF), and the intracellular concentration of HbS. Clinical manifestations of SCD appear to be driven by two major pathophysiological processes: vaso-occlusion with ischemia-reperfusion injury and hemolytic anemia ^13^. Vaso-occlusion occurs because of adhesive interactions of leukocytes and sickle RBCs with the endothelium causing microvascular obstruction and subsequent tissue ischemia ^13^. These episodes of vascular obstruction are followed by the restoration of blood flow, which promotes further tissue injury by reperfusion. The inflammatory cascade resulting from ischemia-reperfusion is amplified by the activation of CD1d-restricted invariant natural killer T (iNKT) cells ^14^. The release of free plasma hemoglobin following intravascular hemolysis results in direct scavenging of nitric oxide (NO), as well as the generation of reactive oxygen species, powerful scavengers of NO ^15, 16^. NO is usually produced by the endothelium and regulates basal vasodilator tone as well as inhibits the activation of platelets and the coagulation system and the transcriptional expression of nuclear factor κB (NFκB)-dependent adhesion molecules, such as vascular cell-adhesion molecule-1, intercellular cell-adhesion molecule-1, and selectins ^17, 18^. HbS polymerization as well as its multiple downstream consequences, including endothelial cell injury, endothelial dysfunction, increased oxidant stress, inflammation, and coagulation and platelet activation, are therapeutic targets in SCD ( Figure 1). SCD has been dichotomized into two overlapping sub-phenotypes: viscosity-vaso-occlusion (higher hemoglobin levels, possibly increased blood viscosity, and complications such as osteonecrosis, acute chest syndrome, and acute pain crisis) and hemolysis-endothelial dysfunction (increased hemolysis with lower hemoglobin levels and higher levels of hemolytic markers, including reticulocyte count and serum lactate dehydrogenase, and complications such as leg ulcers, priapism, stroke, and possibly pulmonary hypertension) ^19^. While somewhat simplistic, this classification is useful to understand the pathobiology of disease complications and may provide guidance on the effects of therapies on disease-related complications. The pathophysiology of SCD has been reviewed more extensively elsewhere ^20– 22^.

## Drug therapies for sickle cell disease

Despite an improved understanding of the pathophysiology of SCD, available drug treatments remain limited. For many years, hydroxyurea was the only drug available to modify the severity of SCD ^23^. However, there has been progress in the development of drug therapies with recent approvals of L-glutamine, crizanlizumab, and voxelotor by the US Food and Drug Administration (FDA) ^24– 26^. Based on our current understanding of SCD pathophysiology, there are multiple possible approaches to treating the disease and its complications, including inhibition of HbS polymerization and amelioration of multiple downstream sequelae of HbS polymerization.

### Inhibition of sickle hemoglobin polymerization

Therapeutic approaches targeting HbS polymerization, including 1) blocking intermolecular contacts in the sickle fiber, 2) induction of HbF synthesis, 3) reduction of intracellular HbS concentration, 4) increase in oxygen affinity, and 5) reduction of the concentration of 2,3-diphosphoglycerate, have been described ^27^. Table 1 lists ongoing studies of anti-sickling agents in SCD.

**Table 1.** Ongoing clinical trials of anti-sickling agents in sickle cell disease.

Mechanism	Drug	Sponsor	NCT number (study acronym)	Clinical phase/status	Intervention	Number/age	Objective
HbF induction	Nicotinamide vs. THU and decitabine	EpiDestiny, Inc; NIH; NHLBI	NCT04055818	Phase I Recruiting	Oral nicotinamide vs. THU + decitabine for 12 weeks followed by combination for a further 12 weeks	20/≥18 years	Compare effect of oral nicotinamide vs. THU-decitabine and in combination on hemoglobin level at week 12
	Panobinostat (LBH589)	Abdullah Kutlar	NCT01245179	Phase I Active, not recruiting	Oral, for 12 weeks	18/≥18 years	Evaluate safety, HbF, F cells, total hemoglobin, markers of inflammation, QOL measures
	Metformin	Baylor College of Medicine	NCT02981329 (FITMet)	Phase I Recruiting	Hydroxyurea + metformin vs. metformin	56/10–60 years	Change in HbF or total hemoglobin, QOL, RNA sequencing
Allosteric modifier (to the R-state)	Voxelotor (formerly GBT440)	Global blood therapeutics	NCT03943615 (expanded access)	Approved for marketing	Oral	≥12 years	To provide early access to patients without alternative treatment options
			NCT04247594	Phase II Recruiting	Oral, open-label	45/18–60 years	Dose escalation study to evaluate safety and tolerability of doses, 1,500 mg to 3,000 mg daily
			NCT02850406	Phase IIa Recruiting	Part A: single dose Part B: 24 weeks Part C: 48 weeks	125/4–17 years	Pharmacokinetics, change in hemoglobin, effect on hemolysis, TCD velocity, safety
			NCT03573882	Phase III Active, not recruiting	Oral, daily	179/≥12 years	Open-label extension study, adverse events, frequency of SCD- related complications
			NCT04218084 (HOPE kids 2)	Phase III Not yet recruiting	Oral voxelotor vs. placebo	224/2–14 years	Evaluate effect on TCD in children
			NCT04188509	Phase III Enrolling by invitation	Oral, open-label	50/4–18 years	Evaluate safety and tolerability, SCD-related complications
Allosteric activator of RBC pyruvate kinase-R	AG-348 (Mitapivat sulfate)	NHLBI	NCT04000165	Phase I Recruiting	Oral, with two dose escalations after 2 weeks	25/≥18 years	Safety and tolerability, pharmacokinetics, and pharmacodynamics
	FT-4202	Forma therapeutics	NCT03815695	Phase I Recruiting	Single and multiple ascending oral doses of FT-4202 vs. placebo	130/12–60 years	Safety, pharmacokinetics, pharmacodynamics
RBC ion transport channels	SCD-101	Invenux	NCT02380079	Phase Ib Recruiting	Part A: open-label, dose- escalation study Part B: randomized, placebo-controlled, cross- over study	60/18–55 years	Safety, change in hemoglobin, markers of hemolysis, QOL measures, functional capacity
	Memantine (NMDAR antagonist)	HaEmek Medical Center, Israel	NCT03247218	Phase IIa/b Recruiting	Oral, once daily for 1 year	40/≥10years	Safety, frequency of hospitalizations, duration of hospitalizations, analgesic use, transfusion requirement, QOL measures

HbF, fetal hemoglobin; QOL, quality of life; NHLBI, National Heart, Lung, and Blood Institute; NMDAR, N-methyl-D-aspartate receptor; RBC, red blood cell; SCD, sickle cell disease; TCD, transcranial doppler; THU, tetrahydrouridine; VOC, vaso-occlusive crisis.

***Induction of fetal hemoglobin.*** High levels of HbF ameliorate the severity of SCD ^8, 28, 29^. Hydroxyurea strongly induces HbF ^30^, although the mechanisms by which it does so are still unclear ^30– 33^. Hydroxyurea improves erythrocyte deformability, lowers hemolysis, decreases circulating leukocytes and reticulocytes ^34^, reduces adhesion receptor expression ^35– 38^, and is an NO donor ^39, 40^. In two placebo-controlled, phase III trials, hydroxyurea significantly reduced the frequency of vaso-occlusive crises, acute chest syndrome, blood transfusion requirement, and hospitalizations in adults and children with SCD ^23, 41^. Treatment with low, fixed weight-based dosing of hydroxyurea (10 mg/kg daily) also decreased the frequency of SCD-related complications with low toxicity ^42, 43^. Hydroxyurea further ameliorates SCD morbidity by decreasing the risk of stroke and chronic kidney disease ^44, 45^ and may reduce mortality in SCD ^46– 49^. Recent studies to optimize hydroxyurea therapy are exploring dose maximization via pharmacokinetics-based dosing ^50– 54^. Despite its multiple benefits, a substantial number of patients on hydroxyurea may not obtain an adequate clinical response.

Inhibitors of epigenetic enzymes which repress γ-globin genes, including DNA methyltransferase (DNMT1), histone deacetylases, and lysine-specific demethylase (LSD-1), and γ-globin gene co-activator agonists are being evaluated in SCD. Decitabine (5-aza-2’-deoxycytidine), a DNA hypomethylating agent which depletes DNMT1, increased HbF and total hemoglobin levels at non-cytotoxic doses in patients who had no responses to hydroxyurea ^55^. However, it has a very short plasma half-life and negligible oral bioavailability due to rapid inactivation by cytidine deaminase (CDA) ^55^. The combination of escalating doses of oral decitabine and an oral CDA inhibitor, tetrahydrouridine (THU), was well tolerated, and the highest tested dose increased HbF, doubled F-cells by approximately 80% of total RBCs, increased total hemoglobin, and decreased biomarkers of hemolysis, coagulation activation, and inflammation ^56^.

Histone deacetylase (HDAC) inhibitors increase HbF levels ^57– 63^. Although treatment with intermittent doses of sodium butyrate produced sustained increases in HbF, F-cells, and total hemoglobin ^57^, the orally available sodium dimethyl butyrate (HQK-1001) did not significantly increase HbF and appeared to result in more pain crisis compared with placebo ^63^. A phase II study of the global HDAC inhibitor vorinostat was terminated early owing to poor accrual. Selective HDAC1/2 inhibition with ACY-957 increased HbF *in vitro* and represents a promising therapeutic approach with a better safety profile ^64^. Based on the success of the LSD-1 inhibitors RN-1 and ORY-3001 in increasing HbF and F-cells in preclinical studies ^65– 67^, INCB059872 was investigated (NCT03132324). However, this phase I study was terminated early for business reasons. Pomalidomide, a third-generation immunomodulatory drug, produced modest increases of HbF, augmented erythropoiesis, and preserved bone marrow function following 8 weeks of treatment in transgenic sickle mice ^68^. Pomalidomide appeared to increase HbF and total hemoglobin only at the highest dose or with greater than 6 months of exposure ^69^. The safety and efficacy of increasing the expression of γ-globin gene co-activators—FOXO3 agonist ^70^ (metformin [NCT02983129]), nuclear factor-like 2 (Nrf2) agonist ^71^ (dimethyl fumarate), and SIRT1 ^72^—to augment HbF levels are being explored.

***Allosteric modification of sickle hemoglobin to its high-oxygen affinity R-state.*** Voxelotor, recently approved under the FDA’s accelerated approval program, is an orally bioavailable small molecule which binds to α-globin chains of hemoglobin, increases hemoglobin oxygen afﬁnity, and stabilizes the oxyhemoglobin state ^73^. Early studies showed a reduction in markers of hemolysis, with a rapid rise in hemoglobin by day 15 ^74^. This response was not accompanied by worsening tissue hypoxia, based on serum erythropoietin levels and change in oxygen consumption (VO _2_ max), even in participants receiving daily doses of 1,000 mg for 28 days and 900 mg for at least 90 days. Furthermore, voxelotor has been shown to reduce blood viscosity *in vitro* ^75^. In a multicenter, phase III study of patients 12 to 65 years of age, randomly assigned to once-daily oral voxelotor doses of 1,500 mg, 900 mg, or placebo, a significantly higher percentage of subjects on 1,500 mg had a hemoglobin response of 1.0 g/dL at 24 weeks compared with placebo ^76^. In addition, subjects on the 1,500 mg dose saw significant reductions in indirect bilirubin and reticulocyte counts from baseline. A dose-dependent increase in hemoglobin following treatment with voxelotor was not accompanied by an increase in pain crisis rate. As this trial was not enriched for subjects with frequent pain crises, appropriately designed studies of patients with frequent pain episodes are needed to determine the effect of voxelotor on reducing such crises. Open-label extension studies to assess the long-term effects of voxelotor are ongoing.

5-hydroxymethyl-2-furfural (5HMF, Aes-103) interacts allosterically with HbS, increasing oxygen affinity and decreasing HbS polymerization and RBC sickling during hypoxia. A phase I trial, with oral doses of up to 4,000 mg, showed no significant side effects ^77^, but a phase II trial was terminated early because of unblinding of drug groups (NCT01987908). FT-4202, a novel selective activator of RBC pyruvate kinase, decreases intracellular 2,3-diphosphoglycerate levels, with a resulting increase in hemoglobin–oxygen affinity. It demonstrated a favorable safety profile in healthy individuals ^78^ and is being investigated in a phase I study in SCD (NCT03815695).

The oxygen delivery therapeutic agents sanguinate (pegylated bovine carboxyhemoglobin) and MP4CO (pegylated human carboxyhemoglobin) are dual transfer agents which release carbon monoxide when delivering oxygen to hypoxic tissues. They stabilize HbS in its R-state and appear to exhibit anti-inflammatory and anti-apoptotic activity by induction of Nrf2 and heme oxygenase-1 ^79, 80^. Sanguinate had an acceptable safety profile ^81, 82^. A phase II study evaluating its efficacy during acute pain crisis was recently completed (NCT02411708).

***Increase red blood cell hydration.*** The inverse relationship between HbS concentration and delay time suggests that even small decreases in intracellular HbS might be beneficial ^83^. Senicapoc selectively blocks the calcium-activated, potassium efflux (Gardos) channel and improves anemia and hemolysis in SCD ^84, 85^. Despite improvements in anemia and hemolysis, a phase III trial showed no significant decrease in the rate of pain crises compared to placebo ^84^. Similarly, studies of agents that block the potassium-chloride co-transport channel showed no clinical benefits ^86– 88^. SCD101, a botanical drug with an unclear anti-sickling mechanism which might involve dilution of HbS by affecting the RBC membrane, was well tolerated in a phase Ib study, with a decrease in chronic pain and fatigue and improvement in leg ulcers ^89^.

### Targeting downstream sequelae of sickle hemoglobin polymerization

***Antioxidant therapy.*** Agents that upregulate antioxidant and/or reactive oxygen species scavenging processes have been evaluated in SCD. Glutamine, a conditionally essential amino acid, is a precursor for nicotinamide adenine dinucleotide (NAD) and improves NAD redox potential. In a randomized, multicenter trial of 230 patients with HbSS or HbSβ ^0^-thalassemia, L-glutamine significantly reduced the number of pain crises, hospitalizations, cumulative hospital days, and frequency of acute chest syndrome compared with placebo ^90^. However, the effect size of L-glutamine in reducing pain crises was relatively small, with only a 25% reduction in the median number of pain crises. L-glutamine was well tolerated, although low-grade nausea, non-cardiac chest pain, fatigue, and musculoskeletal pain occurred more frequently than with placebo. Despite some concerns related to the high dropout rate in the trial (36% L-glutamine arm; 24% placebo arm), L-glutamine was approved by the FDA to reduce the acute complications of SCD in patients 5 years and older ^24^.

In an open-label pilot trial of oral N-acetylcysteine (NAC), treatment with either 1,200 mg or 2,400 mg daily for 6 weeks increased whole blood glutathione levels and decreased erythrocyte outer membrane phosphatidylserine exposure, plasma levels of advanced glycation products, and cell-free hemoglobin in both groups ^91^. A randomized, placebo-controlled, double-blind trial of NAC at 600 mg twice daily for 6 months did not decrease the rate of SCD-related pain days per patient year, vaso-occlusive crises, hospital admission days, number of admissions, or days with home analgesic use compared with placebo ^92^. The safety and efficacy of NAC, administered at a higher dose during pain crisis, is being explored in a phase I/II study (NCT01800526) ( Table 2).

**Table 2.** Ongoing clinical trials of anti-adhesion agents and antioxidants in sickle cell disease.

Mechanism	Drug	Sponsor	NCT number (study acronym)	Clinical phase/status	Intervention	Number/age	Objective
P-selectin antagonist	Crizanlizumab	Novartis Pharmaceuticals	NCT03264989 (SOLACE- adults)	Phase II Active, not recruiting	IV infusion, open-label	57/16–70 years	Pharmacokinetics, pharmacodynamics, safety, and efficacy
			NCT03814746 (STAND)	Phase III Recruiting	IV infusion every 2 weeks for 1 ^st^ month and then monthly for 1 year	240/≥12 years	Compare efficacy and safety of 5 mg/kg and 7.5 mg/kg doses with placebo
			NCT04053764 (STEADFAST)	Phase II Recruiting	IV infusion every 2 weeks for 1 ^st^ month and then every 4 weeks for 51 weeks + SoC vs. SoC alone	170/≥16 years	Evaluating effect on kidney function (albumin-creatinine ratio, protein-creatinine ratio, estimated glomerular filtration rate)
			NCT03938454 (SPARTAN)	Phase II Recruiting	IV infusion every 2 weeks for 1 ^st^ month and then every 4 weeks x 51 weeks	56/≥16 years	Evaluate efficacy in priapism, uncomplicated VOC events
			NCT03474965	Phase II Recruiting	IV infusion every 2 weeks for 1 ^st^ month and then every 4 weeks	100/6 months–<18 years	Evaluate pharmacokinetics, pharmacodynamics, safety, and effect on VOC events
Blockade of fcγrIII receptors	IVIG	Albert Einstein College of Medicine	NCT01757418	Phase I–II Recruiting	Single dose of IVIG vs. placebo given within 24 hours of hospitalization	94/12–65 years	Length of VOC, total opioid use, time to end of VOC, *in vitro* adhesion studies
Antioxidant (increased glutathione)	NAC	Bloodworks	NCT01800526	Phase I/II Enrolling by invitation	IV or oral, NAC Part 1: two doses of IV infusion over 8 hours 4 weeks apart or oral NAC for 4 weeks Part 2 (during VOC): IV infusion every 6 hours for 5 days	20/≥18 years	Evaluate effect on vWF activity, measures of hemolysis and oxidation In Part 2, evaluate efficacy during VOC

IV, intravenous; IVIG, intravenous gammaglobulin; NAC, N-acetylcysteine; SoC, standard of care; VOC, vaso-occlusive crisis; vWF activity, von Willebrand factor activity.

***Anti-adhesive therapy.*** Agents targeting adhesion of blood cells to the endothelium have been investigated in SCD. Crizanlizumab, a humanized monoclonal anti-P-selectin antibody, was recently approved for use in patients 16 years and older for the prevention of vaso-occlusive crises ^25^ based on results of a randomized, double-blind, phase II study which evaluated the benefit of 2.5 mg/kg or 5 mg/kg doses versus placebo. Significantly lower median crisis rate, longer median times to first and second crises, and lower median rate of uncomplicated crises per year were observed with high-dose crizanlizumab compared with placebo following a 52-week treatment period ^93^. Multiple other studies of crizanlizumab are ongoing ( Table 2).

Purified poloxamer 188, a nonionic block copolymer surfactant with hemorheologic and antithrombotic properties, was previously shown to significantly decrease the duration of pain episodes, especially in children and patients on hydroxyurea ^94^. However, a more recent phase III study in children and adults reported no significant effect of purified poloxamer 188 (vepoloxamer or MST-188) on duration of vaso-occlusive crises compared with placebo ^95^. Rivipansel sodium (formerly GMI-1070) is a small-molecule pan-selectin inhibitor that binds to E-, P-, and L-selectin ^96^. In a randomized, double-blind, adaptive, phase II trial, treatment with rivipansel during pain crisis produced a significant reduction in the mean cumulative intravenous opioid analgesic use compared to placebo ^97^. However, despite the promising phase II trial results, the recently completed multicenter phase III RESET trial failed to meet its primary (time to readiness for discharge) and key secondary (time to discharge, cumulative intravenous opioid utilization, and time to discontinuation of intravenous opioids) efficacy endpoints ^98^.

Heparins inhibit adhesive interactions via P-selectin ^99, 100^. Tinzaparin, a low-molecular-weight (LMW) heparin, at therapeutic dose reportedly decreased the number of hospital days, the number of days with pain crisis, and the number of days with the most severe pain scores compared with placebo ^101^. Sevuparin, a derivative of LMW heparin, which retains the P-selectin-binding domain of heparin but largely lacks anticoagulant properties, binds to P- and L-selectins, thrombospondin, fibronectin, and von Willebrand factor, inhibits the adhesion of sickle RBCs to stimulated cultured endothelial cells *in vitro*, and prevents vaso-occlusion with normalization of blood flow in a mouse model of vaso-occlusion ^102^. A recently completed randomized, double-blind, placebo-controlled trial found that, although safe, sevuparin did not significantly reduce the time to resolution of vaso-occlusive crisis ^103^. A phase II feasibility study of therapeutic-dose unfractionated heparin (NCT02098993) was recently terminated for poor enrollment, but a phase III, randomized, placebo-controlled study of tinzaparin in acute chest syndrome is ongoing (NCT02580773).

Intravenous immunoglobulin (IVIG) binds to FcγRIII receptors, inhibits neutrophil adhesion to the endothelium, reduces RBC capture by leukocytes, and reduces Mac-1 activity due to recruitment of Src homology 2-containing tyrosine phosphatase-1 ^104^. IVIG was tolerated at doses of up to 800 mg/kg during acute vaso-occlusive crises and decreased Mac-1 function from baseline ^105^. A phase II clinical trial of IVIG during acute pain episodes is ongoing (NCT01757418).

***Anti-inflammatory agents.*** Multiple approaches to downregulate inflammatory pathways have been evaluated. Initial clinical responses with the use of steroids for pain crisis and acute chest syndrome were followed by “rebound” pain episodes and re-admissions after treatment discontinuation ^106, 107^. Inhaled corticosteroids did not significantly reduce the morbidity of acute chest syndrome in a retrospective cohort study ^108^, but inhaled mometasone significantly reduced daily pain diary scores and levels of soluble vascular adhesion molecule-1 over the 16-week treatment period in another single-center, placebo-controlled trial ^109^.

The activation of iNKT cells is downregulated by the activation of adenosine A2A receptors (A _2A_R) ^110^. Regadenoson, a partially selective A _2A_R agonist, was reportedly safe but did not decrease length of hospital stay, total opioid use, or pain scores compared to placebo during pain crises ^111, 112^. In addition, there were no significant differences between regadenoson and placebo regarding the number of patients exhibiting a greater than 30% reduction in activated iNKT cells. The anti-iNKT cell monoclonal antibody NKTT120 resulted in rapid, specific, and continuous iNKT cell depletion in a single-ascending-dose study ^113^, but further studies are required to determine its long-term safety and efficacy.

Statins have benefits independent of their cholesterol-lowering effect. In a pilot study, simvastatin was well tolerated, increased levels of NO metabolites, and decreased C-reactive protein (CRP) and interleukin-6 levels ^114^. Daily treatment with simvastatin for 3 months significantly reduced pain crises, oral analgesic use, and levels of high-sensitivity CRP, soluble E-selectin, soluble ICAM-1, soluble ICAM-3, and VEGF ^115^, providing supporting data for conduct of a placebo-controlled trial. Atorvastatin was well tolerated in a pilot study but did not improve endothelial function or decrease albuminuria ^116^.

Long-chain omega-3 polyunsaturated fatty acids such as eicosapentaenoic acid (EPA [n-3]) and docosahexaenoic acid (DHA [n-6]) provide benefit to individuals with chronic inflammatory disorders. In a randomized, single-center study, daily omega-3 administered in capsule form significantly lowered the occurrence of vaso-occlusive events, severe anemia, blood transfusion requirements, and school absence compared with placebo ^117^. More recently, a randomized, placebo-controlled study of different doses of SC411, a novel DHA ethyl ester formulation with high DHA bioavailability, found it was well tolerated, with significant reductions in d-dimer and E-selectin and an increase in hemoglobin ^118^. SC411 also significantly reduced electronic diary-recorded pain episodes, analgesic use at home, and days absent from school but did not significantly lower pain crises in the pooled active groups compared to placebo.

Preliminary results suggest that the monoclonal anti-IL1β antibody canakinumab (ACZ885) is well tolerated and not associated with major side effects in SCD ^119^.

***Anticoagulant and antiplatelet therapies.*** With abundant evidence that SCD is a hypercoagulable state ^21^, combined with data that coagulation and platelet activation may play roles in disease pathophysiology ^120– 124^, multiple studies have evaluated the effects of anticoagulants and antiplatelet agents in SCD. Although treatment with the LMW heparin tinzaparin produced more rapid resolution of pain crisis and shorter duration of hospitalization than placebo ^101^, it is uncertain whether the reported beneficial effects were a result of its anticoagulant or anti-adhesive effects. The results of a pilot study of the direct oral anticoagulant rivaroxaban in SCD are awaited (NCT02072668).

Multiple relatively small studies of aspirin have reported modest benefit at best ^125– 127^, although one study reported an increase in oxygen affinity, hemoglobin level, and RBC lifespan ^126^. A pilot study of the glycoprotein IIb/IIIa inhibitor eptifibatide showed that it was safe during acute pain episodes but did not reduce the time to resolution of such episodes ^128^. Ticlopidine, a P2Y _12_ ADP-receptor antagonist, reportedly decreased the number of pain episodes, mean duration of pain episodes, and severity of such episodes ^129^. However, a more recent phase III trial of prasugrel, a newer generation P2Y _12_ receptor blocker, showed no significant reduction of the frequency of pain episodes in children with SCD ^130^. Ticagrelor, a reversible P2Y _12_ receptor blocker, was well tolerated in children ^131^ and young adults ^132^ but had no beneficial effects on pain. A phase III study to determine the efficacy of ticagrelor in reducing vaso-occlusive crises in children is ongoing ^133^ ( Table 3).

**Table 3.** Ongoing clinical trials of anticoagulants, antiplatelet agents, anti-inflammatory agents, and nitric oxide-related drugs in sickle cell disease

Mechanism	Drug	Sponsor	NCT number (study acronym)	Clinical phase/ status	Intervention	Number/age	Objective
Anticoagulant, anti-adhesive (P- selectin blocker)	Tinzaparin (low-molecular-weight heparin)	Assistance Publique –Hopitaux de Paris, LEO pharma	NCT02580773 (TASC)	Phase III Unknown status	Subcutaneous therapeutic vs. prophylactic dose for 7 days	200/≥18 years	Time to ACS resolution, bleeding events, hospital mortality
Antiplatelet	Ticagrelor	AstraZeneca	NCT03615924 (HESTIA3)	Phase III Active, not recruiting	Oral ticagrelor vs. placebo for at least 12 months	193/2–17 years	Evaluate efficacy in decreasing VOC, acute chest syndrome, duration of VOC, QOL measures
Antithrombotic, anti-inflammatory	Defibrotide	New York Medical College	NCT03805581	Phase II Recruiting	Intravenous, every 6 hours during ACS event for maximum of 7 days	20/2–40 years	Safety (allergic reaction, bleeding), improvement in clinical signs of ACS
Anti-inflammatory	Mometasone	Jeffery Glassberg	NCT03758950 (IMPROVE2)	Phase II Recruiting	Inhaled, one puff daily vs. placebo for 48 weeks	80/≥18 years	Change in soluble VCAM-1, hemoglobin, markers of hemolysis, leukocyte count, QOL measures
	Canakinumab (anti-IL1β antibody)	Novartis	NCT02961218	Phase II Active, not recruiting	Subcutaneous, monthly for 6 months vs. placebo, followed by 6-month open-label extension	49/8–20 years	Reduction of average daily pain, days missed from school, blood counts, pharmacokinetics, hs-CRP, measures of hemolysis
	Docosahexaenoic acid and eicosapentaenoic acid	Thomas Jefferson University	NCT01202812	Phase II Not yet recruiting	Oral, daily for 6 months vs. placebo	48/10–19 years	Decrease inflammatory biomarkers, QOL
	Docosahexaenoic acid (SC411)	Micelle BioPharma	NCT02973360 (SCOT study)	Phase II Active, not recruiting	Oral for 8 weeks vs. placebo followed by 49-month open- label extension	68/5–17 years	Safety, pharmacokinetics, effect on VOC
	Omega-3-fatty acid	Sultan Qaboos University	NCT02525107	Phase III Recruitment status unknown	Oral, daily for 52 weeks vs. placebo	280/13–70 years	Effect on VOC, duration of hospitalization, RBC membrane fatty acid profile
	Rifaximin (antibiotic, decrease aged neutrophils)	New York Medical College	NCT03719729	Phase II Recruiting	Oral, twice daily for 1 year	20/18–70 years	Safety, frequency of hospitalizations for VOCs, duration of hospitalizations, RBC transfusions, QOL measures
Increased NO production	Arginine	Emory University	NCT02447874	Phase I/II Enrolling by invitation	Intravenous, three times a day for maximum of 7 days	21/7–21 years	Pharmacokinetics, NO metabolites
			NCT02536170	Phase II Recruiting	Intravenous, three times a day through duration of hospitalization	114/3–21 years	Effect on VOC, including total parenteral opioid use, length of hospital stay, resolution of VOC
Vasodilator	Olinciguat (IW-1701) (soluble guanylate cyclase stimulator)	Cyclerion therapeutics	NCT03285178	Phase II Recruiting	Oral, daily 12 weeks vs. placebo	88/16–70 years	Safety and tolerability, pharmacokinetics, pharmacodynamics
	Riociguat (soluble guanylate cyclase stimulator)	Mark Gladwin	NCT02633397	Phase II Recruiting	Oral, three times daily for 12 weeks	100/≥18 years	Safety, changes in pain intensity, SCD-related complications, functional capacity, blood pressure, plasma NT-proBNP, changes in laboratory measures, TRV
	IMR-687 (selective phosphodiesterase-9 inhibitor)	Imara	NCT03401112	Phase IIa Recruiting	Oral, daily for 24 weeks vs. placebo	70/18–55 years	Safety, pharmacokinetics
			NCT04053803	Phase II Enrolling by invitation	Oral, daily for 49 months, open-label extension	70/≥18 years	Safety, change in hemoglobin, HbF, soluble adhesion markers, renal and cardiac function, QOL
	Ambrisentan (endothelin receptor A antagonist)	Augusta University	NCT02712346	Phase I Active, not recruiting	Oral, once daily for 12 weeks	26/18–65 years	Safety and tolerability, microalbuminuria/proteinuria, TRV, inflammatory markers, pain questionnaire

ACS, acute chest syndrome; HbF, fetal hemoglobin; hs-CRP, high-sensitivity C-reactive protein; NO, nitric oxide; NT-proBNP, N-terminal probrain natriuretic peptide; QOL, quality of life; TRV, echocardiography-derived tricuspid regurgitant jet velocity; VCAM-1, vascular cell adhesion molecule-1; VOC, vaso-occlusive crisis; vWF, von Willebrand factor.

***Nitric oxide and related agents.*** With the role of hemolysis in NO scavenging and subsequent endothelial dysfunction, NO and related agents may be beneficial in SCD ^134^. Inhaled NO did not improve time to resolution of pain crisis, length of hospitalization, opioid usage, or rate of acute chest syndrome compared with placebo ^135^. In another study, inhaled NO did not reduce the rate of treatment failure in adult patients with mild to moderate acute chest syndrome ^136^. However, L-arginine, an NO precursor, significantly decreased total parenteral opioid use and pain scores at discharge compared to placebo in children, although there was no difference in length of hospital stay ^137^. Furthermore, supplementation of adult patients on hydroxyurea with oral arginine significantly increased the level of NO metabolites and reduced the frequency of pain crises versus placebo following 4 months of treatment ^138^. Sildenafil, a phosphodiesterase-5 inhibitor which increases NO-mediated effects by inhibiting the degradation of cyclic guanosine monophosphate (cGMP), was associated with more frequent serious adverse events, predominantly hospitalization for pain, compared with placebo ^139^. No evidence of a treatment effect of sildenafil was seen on evaluated study outcomes. Studies of soluble guanylate cycle stimulators, riociguat (NCT02633397) and olinciguat/IW-1701 (NCT03285178), and the phosphodiesterase-9 inhibitor IMR-687 (NCT03401112; NCT04053803) are ongoing ( Table 3).

## Summary

Recent advances in drug development for SCD raise important issues related to the use and availability of these agents. With limited availability of allogeneic bone marrow transplantation and gene therapy, especially in low- and middle-income countries, the number of recently approved drugs offers hope for further improved outcomes in SCD. Choice of initial drug therapy may be guided by a patient’s clinical sub-phenotype as well as the cost of the drug ( Table 4). Patients with frequent vaso-occlusive complications (acute pain episodes, acute chest syndrome) may benefit from hydroxyurea, L-glutamine, and crizanlizumab, while those with increased hemolytic anemia (or quite possibly complications related to hemolytic anemia) may benefit from hydroxyurea and voxelotor. However, more studies are required before definite recommendations can be made regarding the effect of voxelotor on SCD-related complications. As with other chronic disease conditions, it is important to consider the cost of the drug when deciding on initial therapy. Hydroxyurea is much cheaper than L-glutamine, crizanlizumab, and voxelotor and is likely more cost effective as initial therapy for vaso-occlusive complications and hemolytic anemia. However, with the complex pathophysiology of SCD and the limited clinical efficacy of available pharmacological therapies, it is unlikely that a single drug will ameliorate all SCD-related complications. As such, the availability of multiple drugs offers an opportunity for combination therapy based on different mechanisms of action and non-overlapping side effect profiles. Although more studies are required to evaluate the effect of combinations of these drugs, all of the recently approved agents were tested in combination with hydroxyurea. These studies showed benefit whether or not participants received concomitant hydroxyurea, with no increased toxicity. Despite potential benefits, combination therapy involving the use of multiple drugs increases the likelihood of decreased medication adherence owing to polypharmacy. Like in other chronic diseases, however, this problem may be somewhat ameliorated by the development of pills that contain more than one active drug.

**Table 4.** Summary characteristics of FDA-approved drugs for sickle cell disease.

	Hydroxyurea	L-Glutamine	Crizanlizumab	Voxelotor
Age (years)	≥2	≥5	≥16	≥12
Genotypes	HbSS, HbSβ ^0^ thalassemia	All genotypes (only studied in HbSS, HbSβ ^0^ thalassemia)	All genotypes	All genotypes
Mechanism of action	Multiple, but primarily by increasing HbF production	Uncertain, but thought to reduce NAD redox potential, possible decrease in cell adhesion	Anti P-selectin inhibitor (decreases adhesion of WBCs and RBCs to endothelium)	Decreases HbS polymerization by increasing Hb–oxygen affinity
Route of administration	Oral (capsules/tablets)	Oral (powder)	Intravenous	Oral (tablets)
Clinical effects of therapy	Decreased frequency of VOC, decreased frequency of ACS, decreased hospitalization, decreased RBC transfusion requirement, decreased stroke risk	Decreased frequency of VOC, decreased frequency of ACS, decreased hospitalization	Decreased frequency of VOC	Increased hemoglobin
Effect size for primary endpoint (% change and IRR)	44% decrease in VOC per year (median from 4.5 to 2.5), IRR = 0.56	25% decrease in VOC in 48 weeks (median from 4 to 3), IRR = 0.75	45% decrease in crisis rate per year (median from 3 to 1.6), IRR = 0.55	5.5-fold increase in the hemoglobin responders (9% to 59%) at 24 weeks, incidence proportion ratio = 6.6 *
Common toxicities	Myelosuppression, skin hyperpigmentation, nail discoloration, teratogenicity, decreased sperm counts, nausea and vomiting	Constipation, nausea, headaches, abdominal pain	Nausea, arthralgia	Headache, diarrhea, nausea
Pharmacokinetics	Excreted via kidneys Adjust dose for eGFR <60 mL/min/1.73 m ^2^	Use with caution with hepatic and renal impairment, but no recommended dose adjustment	No dosage adjustments in manufacturer labeling for renal and hepatic impairment (not tested in ESRD)	No dosage adjustment for renal impairment, but not yet studied in ESRD requiring dialysis Dose reduction for severe liver disease (Child Pugh class C)
Cost	$	$$$	$$$$$	$$$$$

ACS, acute chest syndrome; eGFR, estimated glomerular filtration rate; ESRD, end-stage renal disease; FDA, US Food and Drug Administration; Hb, hemoglobin; HbF, fetal hemoglobin; HbS, sickle hemoglobin; IRR, incidence rate ratio; NAD, nicotinamide adenine dinucleotide; RBC, red blood cell; VOC, vaso-occlusive crisis; WBC, white blood cell.

* Patients treated with 1,500 mg of voxelotor had 6.6 times increased proportion of hemoglobin responders (>1 g/dL increase from baseline at 24 weeks)

It is particularly important that ongoing trials of novel drugs for SCD involve individuals in resource-limited countries where the burden of disease is high. This offers patients access to potentially disease-modifying therapies but, perhaps equally as important, contributes substantially to capacity building by improving local infrastructure, strengthening health systems, involving local leaders as collaborators, and training local providers. Furthermore, trials of novel agents in sub-Saharan Africa provide data on the effects of these therapies in a setting with fewer resources and unique challenges, including a high prevalence of malnutrition and infections such as malaria. With the high cost of recently approved agents, mechanisms to ensure affordable access to these drugs should be established. A situation where novel agents are tested in resource-limited countries but patients in these countries are unable to afford such drugs following approval is unacceptable. Finally, studies evaluating the effects of recently approved drugs on organ damage are needed. Demonstration that these agents prevent or decrease end-organ damage would provide additional evidence for the role of drug therapies in improving outcomes in SCD.
